# Psychological Resilience and Career Success of Female Nurses in Central China: The Mediating Role of Craftsmanship

**DOI:** 10.3389/fpsyg.2022.915479

**Published:** 2022-06-29

**Authors:** Huiyuan Xue, Xiaona Si, He Wang, Xiaoren Song, Keke Zhu, Xiaoli Liu, Fen Zhang

**Affiliations:** ^1^Department of Neurology, People’s Hospital of Henan University of Traditional Chinese Medicine, Zhengzhou, China; ^2^Nursing Department of People’s Hospital of Henan University of Traditional Chinese Medicine, Zhengzhou, China

**Keywords:** career success, nurses, psychological resilience, craftsmanship, mediating effect

## Abstract

**Background:**

Nurses’ career success is an important factor affecting the quality of nursing services and the stability of the nursing workforce, and enhancing nurses’ career success level is of key significance to the development of the nursing discipline. As psychological resilience and craftsmanship are important spiritual traits in the process of nurses’ career development, it is important to understand the mechanism of their effects on nurses’ career success level.

**Objective:**

To explore the current situation of craftsmanship, psychological resilience and career success levels of female nurses in central China, and to verify the mediating role of craftsmanship between psychological resilience and female career success using structural equation model.

**Methods:**

A cross-sectional study was conducted among 2359 female nurses from three hospitals in central China through an online questionnaire, including craftsmanship, psychological resilience and career success scale. The data were analyzed by *Z*-test and Spearman rank correlation with SPSS 23.0 statistical software, and the mechanism of the effect of craftsmanship and psychological resilience on career success was completed by AMOS 23.0 statistical software.

**Results:**

The scores of career success, psychological resilience, and craftsmanship of female nurses in central China were 68.00 (61.00, 75.00), 74.00 (64.00, 84.00), and 83.00 (79.00, 95.25). Spearman rank correlation analysis showed that Chinese female nurses’ career success was positively correlated with craftsmanship (*r* = 0.511, *P* < 0.01) and psychological resilience (*r* = 0.595, *P* < 0.01). Craftsmanship played a mediating role between psychological resilience and career success, accounting for 39.3% of the total effect ratio.

**Conclusion:**

The scores of career success and psychological resilience of female nurses in central China are at a moderate level, and craftsmanship plays a mediating role between psychological resilience and career success. It is suggested that nursing managers should pay attention to the importance of career success to nurses’ self-development and nursing team stability, and improve their sense of career success by effectively improving nurses’ psychological resilience and craftsmanship.

## Introduction

Today, with the development of the times and the improvement of human health awareness, people’s demand for health level is increasing day by day, which not only brings new challenges and requirements to the quality of nursing services, but also brings greater professional pressure to clinical nurses, which in turn leads to the increase of nurse turnover rate (Wang et al., 2019). Nursing as an important part of modern medicine, the stability and sustainable development of the nursing team is related to the guarantee of nursing quality (Nibbelink and Brewer, 2018). However, for a long time, the problems such as high turnover rate of nurses and manpower shortages have existed in countries around the world (Wan et al., 2018). According to the survey, the turnover rate of nurses around the world is between 15 and 44%, and especially under the impact of COVID-19, nurses have a higher tendency of stress and burnout than before (Pang et al., 2020; Wang et al., 2022). The high turnover rate and mobility of nurses are not conducive to their own professional growth, and even more so to the stability and development of the nursing community in various countries. In recent years, with the improvement of medical standards, although nursing health care in China has been improved, there are still problems such as high turnover rate and uneven development of nurses compared with foreign countries (Liu et al., 2018; Zhang and Tu, 2020). Therefore, it is of great importance for Chinese clinical nurses to clarify their career development plans, improve their sense of career success so as to reduce their turnover rate.

The sense of career success refers to the positive psychological feelings gradually accumulated by individuals in the course of their career and the related achievements made in their work. It includes both subjective and objective success, and is an important indicator and variable to evaluate individual career development (Seibert et al., 1999; Eby et al., 2003). Currently, studies on career success are mostly focused on the field of management, but in recent years, the number of studies on career fulfillment in the field of nursing field has gradually increased (Wu et al., 2022). Previous studies have shown that career success has a significant positive predictive effect on nurses’ career identity and individual values, which is conducive to motivating nurses’ enthusiasm for work, improve the quality of nursing service, reduce the turnover rate of nurses and stabilize the nursing team (Dan et al., 2018; Zamanzadeh et al., 2019; Wu et al., 2020).

Psychological resilience is a good psychological performance of an individual in the face of adversity, stress or trauma (White et al., 2008). Psychological resilience of nurses refers to the protective resources developed by nurses in their professional experience to safeguard the psychological dynamic balance, and it is a comprehensive psychological process of cognitive, emotional and behavioral tendencies. It is well known that nursing is recognized as a high-stress and high-risk profession, and the tremendous occupational stress and tense doctor-patient relationship not only bring physical and psychological burnout to nurses, but also further deepen the turnover intention of individual nurses (Foster et al., 2020). Research shows that psychological resilience can effectively protect nurses’ psychological boundaries, enabling them to overcome various stresses at work and actively cope with workplace adversities, and that nurses with high psychological resilience have stronger adaptive and regulatory abilities, and will actively seek coping methods in the face of adversity to effectively relieve individual stress and thus achieve their own growth and career development (Rees et al., 2015; Mcdonald et al., 2016).

Craftsmanship, also known as professionalism, spirit at work or vocational culture, refers to the spirit and attitude of individuals in pursuit of excellence and perfection formed in the production process (Kinjerski and Skrypnek, 2004; Sapta et al., 2021). Research shows that individuals with the quality of craftsmanship can not only improve the quality of their products, but also have an advantage in professional competition, thus promoting their career development and success (Kinjerski and Skrypnek, 2008). As a discipline with equal emphasis on knowledge and technology, rigor and science have become the connotation of clinical nurses’ work. Internalizing craftsmanship into all aspects of their work can effectively improve clinical efficiency and nursing service quality (Tanaka et al., 2016; Park and Jung, 2021).

In recent years, with the economic and social development, women’s career development has been valued and extended. However, in the context of Chinese male-centered value system and patriarchal culture, although there is a unified view on the career success of both genders in academic circles, a large number of empirical studies still define career success in terms of male external outcomes, and the research related to women’s own career success is still insufficient (Kirchmeyer, 2002; Xiao and Luo, 2015). Moreover, research has found that nurses’ career success is generally low in the predominantly female Chinese nursing workforce. Therefore it is still necessary to strengthen the research on nurses’ career success (Huang et al., 2019; Wu et al., 2022). In the current nursing practice environment in China, focusing on the career success development of female nurses and seeking their career-family balance can effectively reduce the turnover rate and stabilize the nursing workforce. Therefore, this study proposes the hypothesis that there is a positive association between craftsmanship, psychological resilience, and career success among female nurses, and that craftsmanship has a mediating effect between psychological resilience and career success. By verifying the above hypothesis, it provides a theoretical basis for promoting the career success of female clinical nurses and provides a reference for nurses themselves to reasonably plan their career development and achieve their career success. It also provides a reference for nursing managers to improve nurses’ work enthusiasm and nursing quality, so as to stabilize the nursing team, reduce the turnover rate of clinical nurses, and promote the healthy development of hospitals.

## Materials and Methods

### Study Design and Participants

The study was a cross-sectional survey and 2359 participants were recruited from three tertiary hospitals using convenience sampling method from January to February 2022. The inclusion criteria were: (1) having obtained the qualification certificate of nurses of People’s Republic of China and (2) having given informed consent and voluntarily participating in this study. Nurses’ questionnaires were answered and collected using a network platform method, and the person in charge of the hospital nursing to be surveyed was contacted before the questionnaire was distributed to obtain their cooperation, and the link to the questionnaire was sent to the hospital through the network. The questionnaire was filled out following the principle of informed consent and in an anonymous form, and the method of filling out the questionnaire, the purpose and significance of the study were explained together on the front page of the online questionnaire to ensure that the data was unbiased.

The sample size formula shows that at least 817 nurses are needed for this study, and the sample size is 10 times of the questionnaire entries, which is calculated as *N* = (7 + 19 + 20 + 25)*10 = 710. Considering the invalid and missing questionnaires, the sample size is expanded by 15% based on the original sample size, so at least 817 participants are needed for this study. A total of 2359 questionnaires were collected and 2266 were valid with an effective rate of 96.06%.

### Ethics Statement

The study did not involve human clinical trials or animal experiments, and the investigation was conducted anonymously and voluntarily. The date of the subject was strictly confidential and used only for scientific research, which is in line with the ethical principles of the Declaration of Helsinki, and therefore did not require ethical approval.

### Measures

#### Demographic

Subject demographic characteristics were designed by the researchers, including age (Less than or equal to 25 years old, 26∼30 years old, 31∼35 years old, 36∼40 years old and over 40 years old), education level (Junior college, Undergraduate and Master degree or above), years of experience (Less than or equal to 5 years, 6∼10 years, 11∼15 years and over 15 years), technical title (Primary title, Intermediate title, and Senior title), department type (Internal Medicine, Surgical, Obstetrics and Gynecology, Pediatrics, Outpatient and Emergency, Intensive Care Unit, and Operating Room), relationship status (Married, Single, and Widowed or separate) and positions(Head nurse and Nurse).

#### Women’s Career Success Scale

The Women’s Career Success Scale (WCSS) was developed by Chinese scholar Xiao Wei in 2015 based on relevant theories from home and abroad (Xiao and Luo, 2015). The scale consists of 19 items in 5 dimensions: Intra-organizational career competitiveness (WCSS1, four items), Extra-organizational career competitiveness (WCSS2, four items), Intrinsic satisfaction (WCSS3, four items), Relationship network (WCSS4, three items), and Work-family balance (WCSS5, four items). Items were rated on a 5-point Likert scale, with 1 indicating strongly disagree and 5 indicating strongly agree, and the scale had high content and structural validity. In this study the Cronbach’s alpha coefficient of the scale was 0.927, and its five-dimensional Cronbach’s alpha coefficient ranged from 0.767 to 0.924.

#### Craftsmanship Scale

The craftsmanship scale (CS) is designed to assess the specific values and internal behavioral norms held by individuals at work (Zhao et al., 2020). It consists of five dimensions: Personal growth (CS1), Taking responsibility (CS2), Seeking perfection (CS3), Cherishing reputation (CS4), Determination and persistence (CS5), with a total of 20 items, of which each dimension contains 4 items. The items score from very unimportant to very important on a 5-point Likert scale, with a total score of 100, the higher the score, the higher the level of craftsmanship. In this study the Cronbach’s alpha coefficient of the scale was 0.968, and the Cronbach’s alpha coefficients of the five dimensions ranged from 0.816 to 0.973.

#### Psychological Resilience Scale

The psychological resilience scale (CDRISC) was translated and revised by Xiaonan Yu in 2007 and has been proved to have good reliability and validity in the psychometric measurement of Chinese nurses (Yu and Zhang, 2007). The scale consists of 25 items with 3 dimensions, namely, Tough (CDRISC1, 13 items), Self-improvement (CDRISC2, 8 items), and Optimism (CDRISC3, 4 items). Each item is rated on a 5-point positive scale from 0 to 4, representing “never,” “rarely,” “sometimes,” “often,” and “always.” The higher the total score, the better the psychological resilience. In this study, the Cronbach’s alpha coefficient of the scale was 0.967, and the Cronbach’s alpha coefficients of the three dimensions of resilience, self-improvement, and optimism were 0.937, 0.925, and 0.850 respectively.

### Statistical Analysis

IBM SPSS Statistics 23.0 was used for data statistics and analysis, and descriptive statistics such as frequency and composition ratios were used for analysis of count data, and metrological data of non-normal distribution were described by [*M*(*P*_25_, *P*_75_)]. Independent sample *Z* tests were used to compare statistical differences in career success scores among nurses with different demographic profiles, and the correlation among craftsmanship, psychological resilience, and career success was explored by Spearman rank correlation. AMOS 23.0 statistical software was applied to construct structural equation models to analyze the mechanisms of the effects of craftsmanship and psychological resilience on career success, and Bootstrap test was used to test the significance of the mediating effect, with the number of self-sampling set at 5000, and if the 95% confidence interval did not contain 0, the mediating effect was significant. α was taken as two-sided, and the difference was considered statistically significant at *P* < 0.05.

## Results

### Basic Characteristics

Among the 2266 participants surveyed, female nurses under the age of 40 accounted for 94.17% of the total, and the most of the subjects had worked less than 5 years, of which 90.2% were academic degrees. The univariate analysis of career success scores showed that there was no statistical difference in scores on career success by age and education level (*P* > 0.05), and clinical nurses with administrative positions and senior titles generally scored higher (Table 1).

**TABLE 1:** General information and univariate analysis of career success of female nurses (*n* = 2266).

Variables		*N* (%)	Career success	*Z*	*P-value*
Age (years)				5.218	0.266
	≤ 25	498(21.98)	69.00(61.75,74.00)		
	26∼30	528(23.30)	69.00(62.00,75.00)		
	31∼35	692(30.53)	68.00(61.00,74.00)		
	36∼40	416(18.36)	68.50(61.00,74.00)		
	>40	132(5.83)	69.50(62.00,76.00)		
Educational levels				0.913	0.633
	Junior college	216(9.53)	68.00(60.00,75.00)		
	Undergraduate	2044(90.20)	68.00(61.00,75.00)		
	Master degree or above	6(0.27)	73.00(52.00,75.00)		
Working years(years)					
	≤ 5	852(37.60)	69.00(62.00,75.00)	8.895	0.031
	6∼10	472(20.83)	68.00(61.00,74.75)		
	11∼15	636(28.07)	68.00(60.00,73.00)		
	>15	306(13.50)	69.00(61.00,79.25)		
Job title				6.284	0.043
	Primary title	1580(69.73)	68.00(61.00,74.00)		
	Intermediate title	642(28.33)	68.00(61.00,75.00)		
	Senior title	44(1.94)	72.00(63.00,78.00)		
Department				12.603	0.027
	Internal Medicine	758(33.45)	68.00(61.00,74.00)		
	Surgical	440(19.41)	67.00(60.00,74.00)		
	Obstetrics and Gynecology	128(5.65)	69.00(64.00,75.75)		
	Pediatrics	82(3.62)	70.00(64.00,75.25)		
	Outpatient and other	520(22.95)	68.50(62.00,75.00)		
	Emergency, Intensive Care Unit and Operating Room	338(14.92)	67.00(60.00,72.00)		
Relationship status				6.938	0.031
	Married	1410(62.23)	69.00(61.00,75.00)		
	Single	824(36.36)	68.00(61.00,74.00)		
	Widowed or separated	32(1.41)	61.50(57.50,72.50)		
Positions				–2.627	0.009
	Head nurse	118(5.21)	71.00(63.00,76.00)		
	Nurse	2148(94.79)	68.00(61.00,74.00)		

### Scores of Craftsmanship, Psychological Resilience, and Career Success Among Participants

The results of this study showed that the median total scores of career success, craftsmanship, and psychological resilience of female nurses in central China were 68, 83, and 74 respectively, and the dimension scores of each questionnaire are detailed in Table 2.

**TABLE 2:** Scores of 2266 nurses on each dimension of psychological resilience, craftsmanship, and women’s career success [*M*(*P*_25_, *P*_75_)].

Items	*M*	*P*_25_	*P*_75_	*Minimum*	*Maximum*
Psychological Resilience Score	74.00	64.00	84.00	19.00	100.00
Tough	39.00	34.00	45.00	10.00	52.00
Self-improvement	24.00	20.00	26.00	5.00	32.00
Optimistic	12.00	10.00	13.00	2.00	16.00
Craftsmanship total score	83.00	79.00	95.25	20.00	100.00
Personal growth	16.00	16.00	20.00	4.00	20.00
Taking responsibility	18.00	16.00	20.00	4.00	20.00
Seeking perfection	17.00	16.00	20.00	4.00	20.00
Cherishing reputation	16.00	15.00	19.00	4.00	20.00
Determination and persistence	16.00	15.00	19.00	4.00	20.00
Female Nurse Career Success Score	68.00	61.00	75.00	19.00	95.00
Intra-organizational career competitiveness	14.00	12.00	16.00	4.00	20.00
Extra-organizational career competitiveness	12.00	11.00	14.00	4.00	20.00
Inner satisfaction	16.00	14.00	16.00	4.00	20.00
Relationship network	12.00	12.00	14.00	3.00	15.00
Work-family balance	15.00	12.00	16.00	4.00	20.00

### The Correlation Among Craftsmanship, Psychological Resilience, and Career Success

The results of spearman rank correlation analysis showed that the career success of female nurses in central China was significantly and positively correlated with craftsmanship (*r* = 0.511, *P* < 0.01) and psychological resilience (*r* = 0.595, *P* < 0.01) (Table 3).

**TABLE 3:** Correlation analysis of female nurses’ professional identity with psychological resilience and craftsmanship (*r*, *n* = 2266).

Items	1	2	3	4	5	6	7	8	9	10	11	12	13	14	15	16
(1) CDRISC1	1.000															
(2) CDRISC2	0.843**	1.000														
(3) CDRISC3	0.782**	0.831**	1.000													
(4) CDRISC Total Score	0.965**	0.936**	0.874**	1.000												
(5) CS1	0.468**	0.397**	0.437**	0.462**	1.000											
(6) CS2	0.441**	0.355**	0.404**	0.425**	0.883**	1.000										
(7) CS3	0.462**	0.407**	0.452**	0.461**	0.822**	0.900**	1.000									
(8) CS4	0.343**	0.328**	0.348**	0.353**	0.570**	0.600**	0.660**	1.000								
(9) CS5	0.503**	0.467**	0.499**	0.514**	0.643**	0.643**	0.715**	0.654**	1.000							
(10) CS Total Score	0.511**	0.456**	0.493**	0.513**	0.878**	0.896**	0.910**	0.797**	0.835**	1.000						
(11) WCSS1	0.384**	0.388**	0.372**	0.407**	0.317**	0.272**	0.301**	0.324**	0.364**	0.366**	1.000					
(12) WCSS2	0.185**	0.193**	0.180**	0.197**	0.078**	0.037	0.047*	0.136**	0.142**	0.111**	0.502**	1.000				
(13) WCSS3	0.558**	0.494**	0.517**	0.564**	0.533**	0.489**	0.491**	0.349**	0.516**	0.536**	0.543**	0.331**	1.000			
(14) WCSS4	0.538**	0.470**	0.467**	0.535**	0.589**	0.580**	0.553**	0.389**	0.531**	0.586**	0.448**	0.177**	0.710**	1.000		
(15) WCSS5	0.558**	0.535**	0.540**	0.578**	0.443**	0.394**	0.425**	0.313**	0.500**	0.472**	0.466**	0.219**	0.633**	0.624**	1.000	
(16) WCSS Total Score	0.578**	0.540**	0.542**	0.595**	0.482**	0.429**	0.446**	0.373**	0.519**	0.511**	0.767**	0.600**	0.824**	0.722**	0.778**	1.000

The CDRISC, Psychological Resilience Scale; CDRISC1–CDRISC3 represent the dominant variables of Tough, Self-improvement, and Optimistic; CS, Craftsmanship Scale; CS1–CS5, are Personal growth, Taking responsibility, Seeking perfection, Cherishing reputation, Determination and persistence; WCSS, Women’s Career Success Scale, WCSS1–WCSS5, are Intra-organizational career competitiveness, Extra-organizational career competitiveness, Internal satisfaction, Relationship network, and Work-family balance. *P < 0.05; **P < 0.01.

### The Mediating Effect of Craftsmanship Between Psychological Resilience and Career Success of Female Nurses in Central China

**FIGURE 1:**
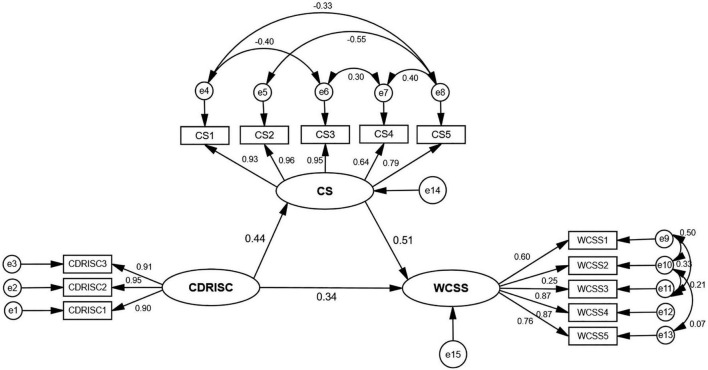
A model of mediating the relationship between psychological resilience and career success among female nurses’ craftsmanship in central China.

Based on the above correlation analysis results and literature review, this study assumes that the structural equation model was constructed with psychological resilience as independent variable, craftsmanship as mediating variable and career success as dependent variable, and confirmatory factor analysis was used to test the fitness of the model. The modified model fit indices were: χ^2^ = 796.208, df = 53, Goodness-of-Fit Index (GFI) = 0.946, Adjusted Goodness-of-Fit Index (AGFI) = 0.907, Standardized Fit Index (NFI) = 0.969, Incremental Fit Index(IFI) = 0.971, Comparative Fit Index (CFI) = 0.971, Tucker-Lewis Index (TLI) = 0.958, Root Mean Square Error of Approximation (RMSEA) = 0.079, Standardized Root Mean Square Residual (SRMR) = 0.0451. The specific model paths, convergent validity and discriminant validity are shown in Tables 4, 5. In this study, the large sample size caused too much freedom in the chi-square values (Xie et al., 2018). Although the chi-square values are not significant, all other indicators meet the requirements, indicating that the model fits well. As shown in Figure 1 and Table 6, the mediating effect value of craftsmanship was 0.138, accounting for 39.3% of the total effect value of 0.353. In summary, craftsmanship plays a part in mediating the effect between psychological resilience and career success among female nurses in central China.

**TABLE 4:** Model parameters of the structural equation for the career success status of female nurses (*n* = 2266).

Path Relationships	Unstd.	*SE*	C.R. (t*-value)*	*P-value*	Std.	CR	AVE
Optimistic **←** Psychological Resilience	1.000				0.904	0.943	0.846
Self-improvement **←** Psychological Resilience	2.111	0.028	74.635	<0.001	0.946		
Tough **←** Psychological Resilience	3.004	0.044	68.529	<0.001	0.909		
Personal growth **←** Craftsmanship	1.000				0.934	0.937	0.751
Taking responsibility **←** craftsman spirit	0.961	0.012	83.509	<0.001	0.964		
Seeking perfection ← Craftsmanship	0.935	0.012	78.443	<0.001	0.953		
Cherishing reputation **←** Craftsmanship	0.658	0.018	37.039	<0.001	0.644		
Determination and persistence **←** Craftsmanship	0.807	0.018	45.896	<0.001	0.793		
Intra-organizational career competitiveness **←** Women’s Career Success	1.000				0.597	0.816	0.499
Extra-organizational career competitiveness **←** Women’s Career Success	0.487	0.039	12.574	<0.001	0.250		
Inner satisfaction **←** Women’s Career Success	1.407	0.043	32.511	<0.001	0.865		
Relationship network **←** Women’s Career Success	1.028	0.035	29.213	<0.001	0.869		
Work-family balance **←** Women’s Career Success	1.487	0.055	27.214	< 0.001	0.757		
Women’s Career Success **←** Craftsmanship	0.340	0.016	20.860	<0.001	0.506		
Women’s Career Success **←** Psychological Resilience	0.214	0.014	15.835	< 0.001	0.344		
Craftsmanship **←** Psychological Resilience	0.407	0.019	21.549	<0.001	0.439		

**TABLE 5:** Structural model discriminant validity.

Variables	AVE	CDRISC	CS	WCSS
CDRISC	0.846	0.920		
CS	0.751	0.439	0.867	
WCSS	0.499	0.566	0.657	0.706

**TABLE 6:** Confidence intervals for structural model mediation effects (5,000 bootstrap samples).

Model path	Estimate	95%CI		*P-value*	Boot SE	Effect ratio (%)
		LLCI	ULCI			
Psychological Resilience **→** Women’s Career Success	0.214	0.179	0.250	0.000	0.018	60.7
Psychological Resilience **→** Craftsmanship **→** Women’s Career Success	0.138	0.120	0.159	0.000	0.010	39.3
Total effect	0.353	0.314	0.389	0.000	0.019	100

## Discussion

### Current Status of Career Success, Psychological Resilience, and Craftsmanship Among Female Nurses in Central China

Nurses’ career success refers to the unity of positive subjective feelings and objective outcomes of clinical nurses in their career (Heslin, 2005). Studies have shown that career success, as a key protective factor for nurses’ career development, has positive effects in reducing nursing brain drain and ensuring the quality of nursing service (Sönmez et al., 2021; Xu et al., 2021). The results of this study show that the median total score of career success of female nurses in central China is 68, which is in the medium level, lower than the results of Dan’s study on career success of nurses in mainland China, indicating that there is still some room for improvement (Dan et al., 2018). The reasons for the low career success of nurses may be related to the low income level, complex working environment and lower social status of domestic clinical nurses (Huang et al., 2019; Wang et al., 2019). In China, while undertaking high-intensity work, clinical nurses have to face the mental stress caused by the increasingly tense doctor–patient conflict. Meanwhile nursing work is only mechanical repetition in the cognition of most people in China is that, far lower than the social status of doctors. Therefore, it is still important to actively explore the current situation and influencing factors of female nurses’ career success and improve their career success level.

In this study, it can be found that there are significant differences in demographics in terms of nurses’ years of work, title, administrative position, marital status, and department. As can be seen in Table 1, the career success scores of nurses with advanced titles are significantly higher than those nurses with other titles, which may be related to the fact that nurses with advanced titles are more mature and stable in terms of work experience, knowledge, and technical level compared to nurses with other two titles (Sönmez et al., 2021). Moreover, with the improvement of titles, the salary and competitiveness of nurses within the organization will be further improved, which is more conducive to their career development and therefore they have higher career success scores. In the score of career success of administrative position, it can be found that nurses with administrative positions have better scores than general nurses (Wu et al., 2022). This may be due to the fact that head nurses, as management staff, have a certain level of status in the hospital and have the opportunity to participate in hospital policy-making, and that nurses with administrative positions have relatively more time and opportunities to study and pursue further training, so as to improve their career success. In addition, previous studies have shown that the older the nurses are, the greater their sense of professional success (Asegid et al., 2014). However, the statistics of this study showed that there was no significant difference in the career success among female nurses of different ages, which may due to the balanced number of nurses in the four age groups of 20–40 included in this survey. In Notzer’s study, it was concluded that nurses with higher education tended to have higher levels of career success (Notzer et al., 2004), and the results of Osujj’s study were the opposite (Osuji et al., 2014). However, in the present study it can be found that there is no significant difference between nurses’ education level and career success, and the analysis may be due to the fact that 90.2% of the subjects in this survey had a bachelor’s degree in education. The findings of effect of education and age on nurses’ career success differed in different studies, so further verification about the effect of age and education on career success needs to be done in future studies. In conclusion, nurses’ career success is influenced by title, administrative position, years of experience and marital status, and a good level of career success for nurses can effectively stabilize the nursing workforce and improve the quality of nursing services. Therefore, nursing managers should focus on the career development of female nurses with low seniority and low titles and strengthen theoretical and skill training for young nurses, encouraging them to actively participate in various academic activities, so as to improve their intra-organizational competitiveness and help them to achieve career success.

From Table 2, it can be found that the median total score of psychological resilience of female nurses in central China is 74, which is at a medium level and is consistent with the results of Klink’s study (Kılınç and Sis Çelik, 2021). Analyzing the reasons, we can see that nurses, as an important group in the hospital, work with high intensity and heavy load, and in this survey the subjects were all female, who compared to male nurses, need to balance their family while completing their work in the hospital within limited time and energy, and the continuous stressful state will not only lead to their negative emotions, but also is not conducive to individual psychological health development. Especially in the current COVID-19, the nursing groups, as one of the main forces to fight against the epidemic, is facing the epidemic directly and at the same time is under great physical and mental pressure (Karabulak and Kaya, 2021; Wahlster et al., 2021). Therefore, in view of the current situation of nurses’ psychological resilience, nursing managers should actively pay attention to nurses’ psychological status and enhance their anti-frustration ability through flexible scheduling, organizational training and psychological guidance. Nurses can achieve family-work balance and appropriate decompression by actively improving their personal knowledge, professional ability and spiritual strengths to maintain optimal physical and mental health.

The findings of this study show that female nurses in central China have a high level of craftsmanship. Exploring the reasons can know that, as nursing is a discipline with equal emphasis on knowledge and technology coupled with the particularity of clinical work in hospitals, nurses need to maintain operational rigor and science. Meanwhile, due to the renewal and supervision of medical and health institutions, nurses must continue to study and maintain rigor in order to ensure the precise completion of their work, which coincides with the craftsmanship of excellence, preciseness and truth-seeking (Park and Jung, 2021). And for women in China, compared to other professions, nursing has relative stability which is in line with most women’s pursuit of work. At the same time, since all the nurses included in this study were women, compared to male nurses, women are naturally meticulous and cautious, and therefore have a higher level of craftsmanship score.

### Psychological Resilience, Craftsmanship, and Career Success Correlation Among Female Nurses in Central China

The results of this study showed that the psychological resilience of female nurses in central China was positively correlated with career success (*r* = 0.595, *P* < 0.01), and the better their psychological resilience, the higher their level of career success. In clinical work, nurses with higher psychological resilience tend to have higher enthusiasm and initiative in work and can deal with emergencies in clinical work actively and effectively. Nurses with higher psychological resilience can effectively maintain individual physical and mental balance when suffering from adversity and stress, and have stronger resilience and rebound ability, so that they are more likely to be competent in their jobs and achieve career success (Kim and Yoon, 2018; Foster et al., 2019). Therefore, nursing managers should pay attention to nurses’ psychological state, create a good working atmosphere for them, actively guide them to change their psychological resilience, and finally improve their sense of professional success. In this study, it can be found that nurses’ craftsmanship is also positively correlated with their career success (*r* = 0.511, *P* < 0.01), that is, nurses with high levels of craftsmanship are more likely to achieve career success. In clinical work, nurses with a high level of craftsmanship can more deeply appreciate the meaning of nursing work and reflect the rigor and scientific nature of nursing work. They are willing to devote more energy and emotion than other nurses to continuously pursue individual breakthroughs and work excellence in their nursing career, so as to gain higher recognition and promotion and achieve career success. Therefore, in the career development of nurses, managers should not only focus on the personal growth of nurses, but also pay more attention to the development of their professionalism, so as to improve their sense of career success by enhancing their inner craftsmanship. Moreover, by mastering the correlation between nurses’ professional success, psychological resilience and craftsmanship, nursing managers can more effectively carry out reasonable career planning and development for nurses.

### Craftsmanship Partially Mediates the Relationship Between Psychological Resilience and Professional Success Among Female Nurses in Central China

The results of the mediating effect analysis showed that craftsmanship partially mediated the relationship between psychological resilience and career success among female nurses in central China, with the mediating effect value of craftsmanship being 0.138, accounting for 39.3% of the total effect, indicating that nurses’ career success can be predicted not only by psychological resilience directly, but also by this mediating variable of craftsmanship level. Nurses with higher psychological resilience have better work stability and motivation, and are not only better able to adapt to their work, but also more able to actively regulate their own state and rationally seek solutions when facing difficulties and pressures at work, so as to achieve better work results and career success (Xu et al., 2021); and for individual nurses, a better craftsmanship enables them to seek higher professional values in the work process, exercise and develop their personal abilities while completing their work, and thus achieve their professional goals and values (Jang et al., 2016). Thus, it shows that the craftsmanship, psychological resilience and career success of female nurses play a positive role, and craftsmanship has a significant mediating effect between psychological resilience and career success. The verification of the above model hypotheses further provides a theoretical basis for promoting the career success of female clinical nurses. Therefore, it is suggested that nursing managers should actively pay attention to the relationship between nurses’ psychological resilience, craftsmanship and career success, make full use of internal hospital resources, and develop psychological aids that are in line with the actual situation of nurses in hospitals as a whole, so as to enhance nurses’ psychological resilience. In daily work, hospitals should do a good job in on-the-job training for nurses to enhance their professional identity and confidence, stimulating their love and persistence for their work, so as to improve their sense of career success.

### Limitations

This study still has some limitations. Firstly, this study was conducted in the form of a self-report questionnaire, which is somewhat subjective; secondly, only three tertiary hospitals in central China were selected for the survey, and the sample may have been geographically diverse; finally, due to limited funding, only convenience sampling was used in the study, and only a cross-sectional study was conducted, failing to conduct a random sampling and intervention study based on demographic information. Therefore, in future studies, we will use a stratified sampling method to conduct a multi-regional, multi-center study in China to obtain more scientific findings and provide a basis for further intervention studies on the career success of female nurses in the future.

## Conclusion

In this study, we conducted a cross-sectional survey on career success, psychological resilience and craftsmanship of female nurses in central China through a convenience sampling method in an attempt to understand the level of scores and the structural model relationship among the three. Finally, we found that their psychological resilience and career success were at moderate levels, psychological resilience and craftsmanship had positive predictive effects on the level of career success, and craftsmanship played a partly mediating role between psychological resilience and career success. Therefore, proper psychological and craftsmanship-based interventions should be provided to female nurses to enhance their frustration resistance and craftsmanship in the practice setting, and improve their career success by increasing the level of psychological resilience and craftsmanship. At the same time, it was suggested that nursing managers should actively pay attention to changes in the psychological and career success levels of female nurses, help them to plan their career development rationally and cultivate their craftsmanship by organizing career planning training and creating a positive work environment, so as to improve their career success, reduce the turnover rate and enhance nursing quality, achieving the goal of promoting healthy hospital development. these findings may provide effective references for further improvement of nurses’ career success in the future.

## Data Availability Statement

The original contributions presented in this study are included in the article/Supplementary Material, further inquiries can be directed to the corresponding author.

## Author Contributions

HX, XSi, and FZ completed the conception and design of the study. XSo and KZ performed the data collection and collation. HX and XSi wrote the first draft. FZ, HW, and XL performed the manuscript revision. HX completed the statistical analysis. All authors actively participated in the study and reviewed and agreed on the final manuscript.

## Conflict of Interest

The authors declare that the research was conducted in the absence of any commercial or financial relationships that could be construed as a potential conflict of interest.

## Publisher’s Note

All claims expressed in this article are solely those of the authors and do not necessarily represent those of their affiliated organizations, or those of the publisher, the editors and the reviewers. Any product that may be evaluated in this article, or claim that may be made by its manufacturer, is not guaranteed or endorsed by the publisher.
